# The importance of PSMA PET/CT in evaluating biochemical recurrence in patients with prostate cancer and the need to expand access to this examination via public health care systems

**DOI:** 10.1590/0100-3984.2024.57.e5-en

**Published:** 2024-09-23

**Authors:** Ronaldo Hueb Baroni

**Affiliations:** 1 Hospital Israelita Albert Einstein, Faculdade Israelita de Ciências da Saúde Albert Einstein, Colégio Brasileiro de Radiologia e Diagnóstico por Imagem. São Paulo, SP, Brazil. Email: rhbaroni@gmail.com

Prostate cancer is a highly prevalent neoplasm, with an estimated 288,300 and 71,730 new cases occurring in the United States and Brazil, respectively, in 2023^(1,2)^. That high prevalence results in high health costs for the population, whether in the diagnosis, treatment, or follow-up of the affected patients. In recent decades, great progress has been made toward promoting the diagnosis of prostate cancer in its earlier stages (thus reducing morbidity and mortality). For example, the use of prostate-specific antigen (PSA) testing has become widespread, as has, more recently, multiparametric magnetic resonance imaging to stratify the risk of clinically significant neoplasms and to guide biopsies^(3)^. Nevertheless, up to 40% of patients undergoing radical prostatectomy and up to 50% of patients undergoing radiotherapy will develop biochemical recurrence within the first 10 years after the treatment^(4-6)^.

The advent of gallium-68-prostate-specific membrane antigen positron emission tomography/computed tomography (^68^Ga-PSMA PET/CT), in the early 2010s, represented a true paradigm shift in the evaluation of biochemical recurrence after prostate cancer treatment. Since then, hundreds of studies have been carried out, initially experimental and retrospective, followed by prospective studies, mainly in centers in Europe and subsequently in other countries around the world, including Brazil^(7)^. In 2015, the largest prospective series ever published showed that ^68^Ga-PSMA PET/CT had a sensitivity of 76.6%, specificity of 100%, positive predictive value of 91.4%, and negative predictive value of 100% for the evaluation of lesions in patients with biochemical recurrence^(8)^. In 2016, a systematic review and meta-analysis demonstrated that ^68^Ga-PSMA PET/CT positivity increased in parallel with increasing serum PSA levels in cases of biochemical recurrence, reaching 95% positivity in patients with a PSA > 2 ng/mL^(9)^. Despite these extremely impressive results, it took a long time for PSMA PET/CT to gain approval and wider clinical use in several countries, mainly in public health care systems, because of the cost of producing the radiopharmaceutical and the limited availability of PET equipment, among other factors.

The work conducted by Bogoni et al.^(10)^, recently published in Radiologia Brasileira, highlights an important aspect related to the use of PSMA PET/CT in public health care facilities in Brazil. Although the authors found that the use of the examination in patients with biochemical recurrence has resulted in an increase in the cost per patient in comparison with the standard investigation strategy (without the use of PSMA PET/CT), they also noted a change in the treatment strategy in a substantial proportion (61%) of the patients, with a reduction in the number of futile/inappropriate salvage therapies. That could reduce the overall cost of treating such patients and enable better allocation of resources (for example, to PSMA PET/CT itself).

Cost-effectiveness studies regarding PSMA PET/CT are still scarce because of the short time that the radiopharmaceutical has been in clinical use. The cost-effectiveness of PSMA PET/CT in the primary staging of prostate cancer has already been evaluated in a few studies, the main one being a multicenter, prospective, randomized study^(11)^, in which PSMA PET/CT was shown to have comparatively lower direct costs, as well as greater accuracy, in relation to conventional diagnostic methods (computed tomography and bone scintigraphy).

We hope that studies like those cited above will become increasingly more common. Such studies will enable better allocation of resources and the development of guidelines for access to supplies and diagnostic tests via public health care systems that are more appropriate, especially in lowto middle-income countries like Brazil.
